# *Allamanda cathartica*: A Review of the Phytochemistry, Pharmacology, Toxicology, and Biotechnology

**DOI:** 10.3390/molecules24071238

**Published:** 2019-03-29

**Authors:** Vera L. Petricevich, Rodolfo Abarca-Vargas

**Affiliations:** Facultad de Medicina de la Universidad Autónoma del Estado de Morelos (UAEM), Calle, Leñeros, esquina Iztaccíhuatl s/n. Col. Volcanes, Cuernavaca, C.P. Morelos 62350, Mexico; vera.petricevich@uaem.mx

**Keywords:** *Allamanda cathartica*, phytochemistry, pharmacology, toxicology and biotechnology

## Abstract

In this work, we explore the current knowledge about the phytochemistry and in vitro and in vivo evaluations of the extracts and, where appropriate, the main active components characterized and isolated from the *Allamanda cathartica*. Of the 15 *Allamanda* species, most phytochemical, pharmacological, and toxicological studies have focused on *A. cathartica*. These plants are used for the treatment of various health disorders. Numerous phytochemical investigations of plants from the *A. cathartica* have shown the presence of hydrocarbons, alcohols, esters, ethers, aldehydes, ketones, fatty acids, phospholipids, volatile compounds, phenolic compounds, flavonoids, alkaloids, steroids, terpenes, lactones, and carbohydrates. Various studies have confirmed that extracts and active substances isolated from the *A. cathartica* have multiple pharmacological activities. The species *A. cathartica* has emerged as a source of traditional medicine used for human health. Further studies on the phytochemical, pharmacological, and toxicological properties and their mechanisms of action, safety, and efficacy in the species of *A. cathartica* is recommended.

## 1. Introduction

The plant *Allamanda* is a very widespread group throughout the world. It belongs to the family Apocynaceae and, according to the “The Plant List,” contains approximately 15 species (*A. augustifolia*, *A. blanchetti*, *A. caccicola*, *A. cathartica*, *A. doniana*, *A. laevis*, *A. martii*, *A nobilis*, *A. oenotherifolia*, *A. polyantha*, *A. puberula*, *A. schottii*, *A. setulosa*, *A. thevetifolia*, and *A. weberbaueri*) [1]. The objective of this work is to present complete information about the current research on the distribution, phytochemistry, pharmacology, toxicity, and biotechnology of *Allamanda cathartica*; to identify its therapeutic potential; and to direct future research opportunities. The most relevant data were searched using the keyword “*Allamanda cathartica*” in “Google Scholar”, “PubMed”, “ScienceDirect”, “Scopus”, “Taylor & Francis”, “Web of Science”, and “Wiley”. The taxonomy was validated using the “The Plant List”.

## 2. Ethnobotany

### 2.1. Botanical Characterization

**Figure 1:**
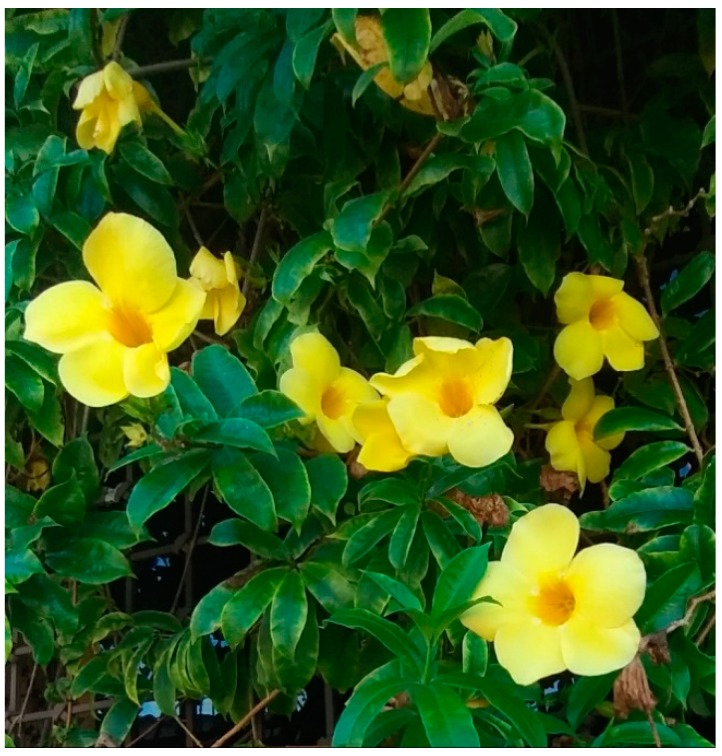
*Allamanda cathartica*.

The genus *Allamanda* is endemic to South America [2]. The genus is named after the Swiss botanist Jean Frédéric-François Louis Allamand, who collected seeds in Suriname and sent them to Carlos Linnaeus to be named in 1771 [3]. *A. cathartica* plants are robust shrubs growing up to 6 m tall. The leaves are elliptical to obovate, opposite, or in whorls. The flowers are yellow and trumpet-shaped, with corolla tubes. The flowers are similar in size to the leaves. The fruits are capsules with spins, and the seeds are compressed and winged. The shrubs, with their beautiful yellow flowers, are popular ornamentals [4]. The species flowers grow all year round, and fruits grow from April to July and in October. In botanical texts, *A. cathartica* is reported to have a wide global distribution in warm climates (Figure 1) [2]. Based on these data, a more exhaustive analysis of the scientific literature was performed.

### 2.2. Distribution

*A. cathartica* plants are distributed in tropical and subtropical areas of many countries, including the United States, México, Belize, Honduras, Nicaragua, Costa Rica, Panama, Venezuela, Bolivia, Ecuador, Guyana, French Guyana, Paraguay, Peru [2], Guatemala [5], El Salvador [6], Puerto Rico [7], Trinidad and Tobago [8], Surinam [9], Cuba [10], Martinique [11], Colombia [12], Brazil [3], Hawaii [13], India [14], the Andaman islands [15], Bangladesh [16], Pakistan [17], Malaysia [18], Indonesia [19], The Philippines [20], Thailand [21], Singapore [22], Hong Kong [14], Myanmar [11], Nepal, Sri Lanka [23], China [24], Australia [25], Kuwait [26], Ghana [18], the Republic of Mauritius [27], Cameroon, Madagascar [2], Nigeria [28] Zimbabwe [29], and France [20].

### 2.3. Synonyms

Synonyms of *Allamanda cathartica* include *Echites verticillata* Sessé and Moç, *Orelia grandiflora* Aublet, *Allamanda grandiflora* (Aublet) Poiret in Lam, and *Allamanda hendersonii* W. Bull ex Dombrain [30], as well as *Allamanda schotti* (Pohl) [31]. In the various countries where *Allamanda* is found, other popular names have been attributed to it.

The following are synonyms: (in Australia) Allamanda [25]; (in Bangladesh) Allamanda [32], Allokananda [23], and Fok Kaia [33]; (in Brazil) Buiussu, Carolina [34], Alamanda, Cipó-de-leite, Dedal-de-dama, Alamanda-amarela, Alamanda-de-flor-grande, Guissú, Quatro-patacas-amarelas [35], Golden trumpet, Yellow Bell, and Buttercup flower [30]; (in Cuba) Flor de barbero, Barbero loco, Flor de mantequilla, Jazmín de la tierra [10], and Jazmín de Cuba [36]; (in El Salvador) San José [6,37]; (in France) Jasmin dÁmarilla [20]; (in French Guiana) Orélie de la Guyana [20]; (in Guatemala) Amanda, Butter cup, and Campana [5]; (in Hawaii) Lani-ali’I and Allamanda [13]; (in India) Jaharisontakka, Pilikaner, Pivikanher [20], Almanda, golden trump vine, [38], Haldhia phool [39], Ghonta phool [40], and Golden trumpet [41]; (in Indonesia) Bunga Terompet [16]; (in Malaysia) Jamaican sunset [42]; (in Mexico) Berta, Cuernos de chivo, Chicliyo [2], and San José [6,37]; (in Nigeria) Allamonda, Yellow allamanda, Golden trumpet [43], Nkutu [44], and Ako-dodo [45]; and (in Thailand) Golden trumpet [21].

### 2.4. Traditional Medical Use

In traditional medicine, *A. cathartica* is indicated for various treatments in many parts of the world: as an antifungal (United States, Caribe [3], and Bangladesh [23]), antiviral (United States and Caribbean [3]), anticancer (Malaysia [46]), and cathartic (India [20] and Bangladesh [23]) or to treat colic (India [47]) or diabetes (India [48]). It is also used as a diuretic and an emetic (India [38]); for the treatment of fever (India [39] and Brazil [34]), hydragogue ascites (India [20] and Bangladesh [23]), hypertension (the Philippines [49] and Bangladesh [23]); to improve blood circulation (Indonesia [16]); and to reduce inflammation (Nigeria [43]). It is also used to treat jaundice (Suriname [8], Brazil [34], and Malaysia [46]), laxative (India [38], Suriname [8], and Nigeria [44]), and Malaria (Nigeria [45], Suriname, [8], Philipphines [20], Malaysia [46], and Brazil [34]). The milky sap is used for lead colic (Mexico and El Salvador [36]), parasitosis (Brazil [34]), rheumatism (Bangladesh [33]), scabies and lice elimination (Brazil [34]), snake bites (Bangladesh [23], Colombia [12], and India [20]), and splenomegaly (Suriname [8] and Brazil [34]). The plant parts used most frequently, in decreasing order, are the leaves, stem bark, flowers, roots, stem, sap, seeds, and branches.

## 3. Phytochemistry

The chemical constituents of *A. cathartica* have been extensively studied since 1954 [14]. Preliminary chemical studies showed the presence of alkaloids [13], anthraquinones [50], anthocyanins [51], carbohydrates [52], carotenoids [21], coumarin [53], flavonoids [54], glycosides [28], hydrocarbon [52], lignin [51], lipids [50,52], phenolic compounds [54], quinones [53], saponins [28,54], steroids [54], tannins [28,54], and terpenes [53,54] from various extracts, mainly leaves, flowers, stems, stem bark, roots, and shoots.

Only these groups of chemical compounds have been isolated and identified, and no anthraquinones, anthocyanins, coumarin, quinones, or lignins have been found. The Marvin program was used to draw the structures of organic chemical compounds [55].

In an analysis of the inorganic composition by atomic absorption spectrophotometry from flowers, the following elements were detected at the following concentrations: Fe (12.21 ± 0.038 µg/g), Mn (1.338 ± 0.049 µg/g), Ni (0.593 ± 0.014 µg/g), Cu (0.348 ± 0.006 µg/g), Cr (0.181 ± 0.032 µg/g), Pb (0.104 ± 0.024 µg/g), and Co (0.089 ± 0.010 µg/g) [56].

### 3.1. Hydrocarbons

**Figure 2:**
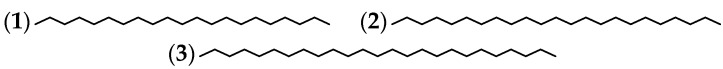
The structures of the hydrocarbons from *A. cathartica*.

The presence of 3 hydrocarbons has been confirmed in *A. cathartica* flowers (Table 1 and Figure 2).

### 3.2. Alcohol, Ester, Ether, Aldehyde, and Ketone

**Figure 3:**
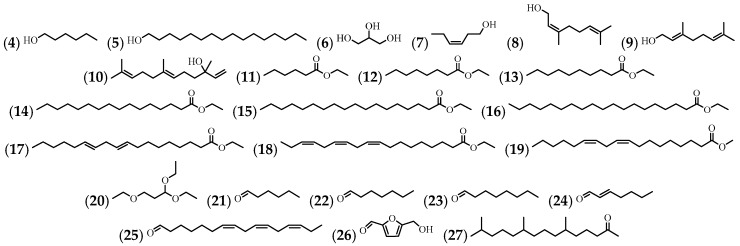
The structures of the alcohols, esters, ethers, aldehydes, and ketones from *A. cathartica*.

Seven alcohol compounds were identified, as well as 9 esters, 1 ether, 6 aldehydes, and 1 ketone in various extracts of flowers, leaves, and stems (Table 2 and Figure 3).

### 3.3. Fatty Acids and Phospholipids

**Figure 4:**
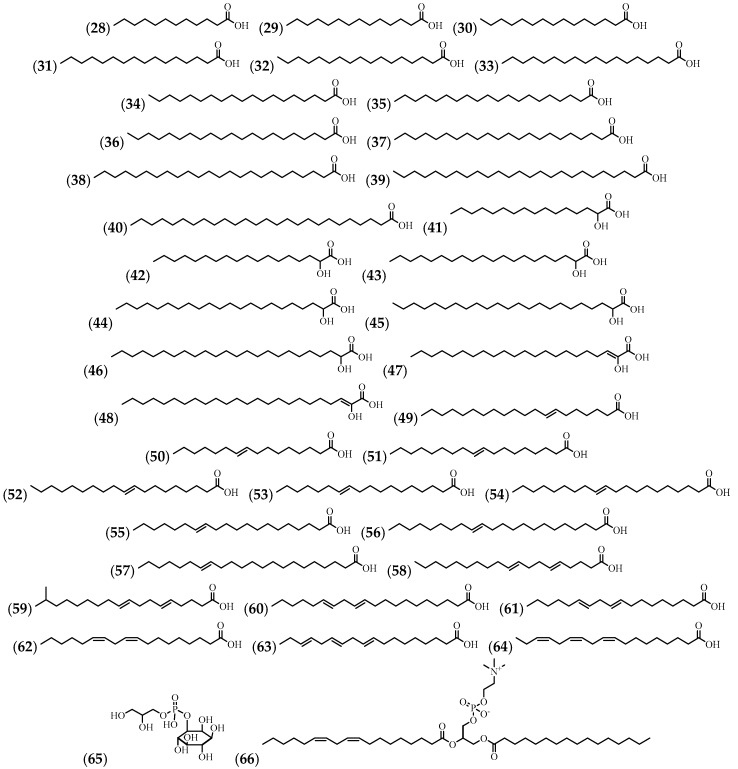
The structures of the fatty acids and phospholipids from *A. cathartica*.

A fatty acid composition analysis resulted in the identification of 37 compounds and a compound of very unusual structure (**59**). Two phospholipids were also identified. The flowers, leaves, and stems were used for the isolation of these compounds (Table 3 and Figure 4).

### 3.4. Volatile Compounds

**Figure 5:**
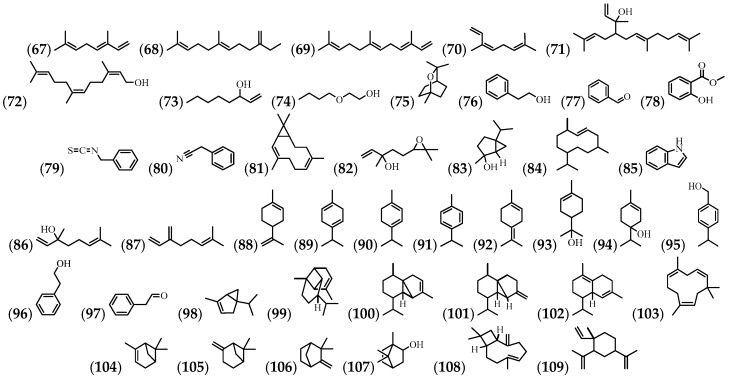
The structures of the volatile compounds from *A. cathartica*.

A total of 43 volatile compounds have also been identified, mostly in flowers and leaves (Table 4 and Figure 5).

### 3.5. Phenolic Compounds and Flavonoids

**Figure 6:**
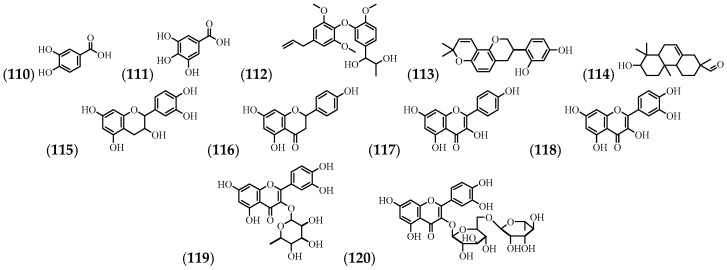
The structures of the phenolic compounds and flavonoids from *A. cathartica*.

From the flowers and stems, 5 phenolic compounds and 6 flavonoids have been identified (Table 5 and Figure 6).

### 3.6. Alkaloids

**Figure 7:**
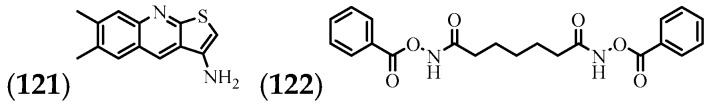
The structures of the alkaloids from *A. cathartica*.

Two alkaloids present in the stems are the only ones reported in the literature [38] (Table 6 and Figure 7).

### 3.7. Steroids and Terpenes

**Figure 8:**
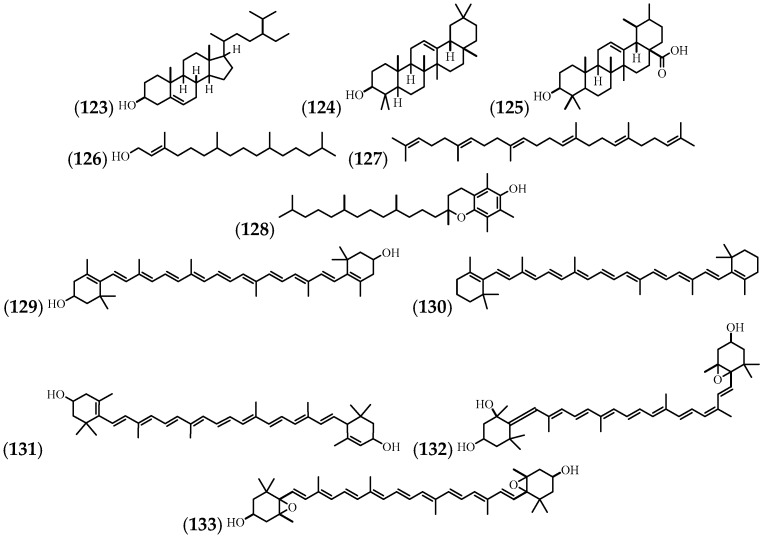
The structures of the steroids and terpenes from *A. cathartica*.

Carotenoids are terpene compounds. They can be yellow, orange, or red in pigment, and they are widely distributed in nature. In plants, they play an important role in photosynthesis and in the colouring of flowers and fruits [62]. *A*. *cathartica* carotenoids have been found in flowers, leaves, and stems (Table 7 and Figure 8).

### 3.8. Lactones

**Figure 9:**
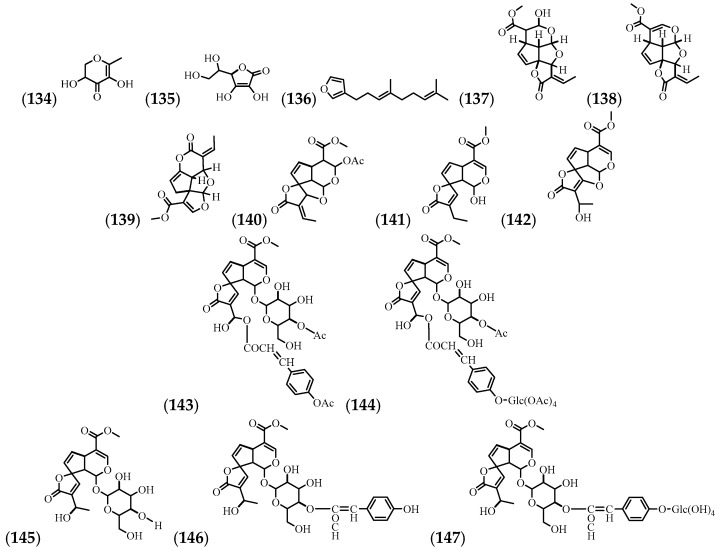
The structures of the lactones from *A. cathartica*.

The mechanisms for recovering compound (**145**) from ethanol and ethyl acetate extracts have been established, with ethanol showing the greatest yield [64]. The most commonly used plant parts for the isolation and identification of compounds are flowers, roots, leaves, root bark, and bark (inner part) (Table 8 and Figure 9).

### 3.9. Carbohydrates

**Figure 10:**
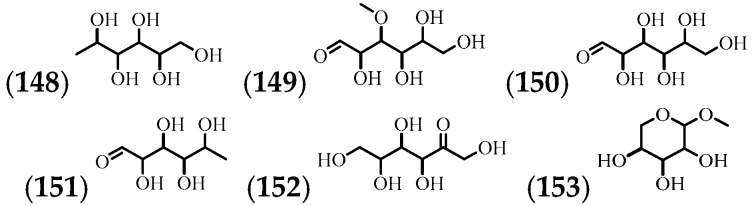
The structures of the carbohydrates from *A. cathartica*.

The presence of 6 carbohydrates in the leaves, stems, and nectar has been shown (Table 9 and Figure 10).

## 4. Pharmacological Activity

*A. cathartica* has been reported in traditional medicine, and the first biological and pharmacological studies were documented in 1943 [68]. A more general view of the pharmacological investigations on various crude extracts and isolated chemical compounds of the species are described below.

### 4.1. Analgesic

In a previous study conducted in our laboratory, it was observed that the ethanol extract from the aerial parts of *A. cathartica* showed an analgesic activity in the murine model.

### 4.2. Anti-Inflammatory

The inhibition of haemolysis in human erythrocytes by an aqueous fraction from a methanol extract was evaluated, with rates of 69.49 ± 0.49% compared to the positive control acetyl salicylic acid (0.1 mg/mL), which showed a 72.79% inhibition [69]. In another study, the compound (**119**) obtained from fresh *A. cathartica* flowers was evaluated for anti-inflammatory activity using an in vitro haemolytic membrane stabilization study. The effect of inflammation was studied using erythrocytes exposed to a hypotonic solution. The results indicated that the obtained compound showed a membrane stabilizing activity, which was highest with 75 µg [70]. In an in vivo model, the compound (**145**) from a flower ethanol extract was evaluated for activity against ulcerative colitis induced by dextran sulfate sodium (DSS) in female mice. As a standard control, 5-Amino-Salicylic Acid was used, and the mice were administered either compound at the same dose (100 mg/kg/day for 7 days). Treatment with the (**145**) compound resulted in less shortening of the colon, improved histological damage, and less mucin depletion of the intestinal mucosa compared to the group only treated with the vehicle [71].

### 4.3. Antidepressant

The antidepressant activity of the compound (**145**) was evaluated in Swiss Webster female mice (0.5, 1, and 2 μg/kg i.p). Doses of 1 and 2 μg/kg showed a significant difference *p* < 0.001 with respect to the negative control. Imipramide (20 mg/kg i.p.) was used as a positive control [61].

### 4.4. Antidiabetic

Aqueous extracts from the aerial parts of *A. cathartica* (400 mg/kg for 28 days) reduced blood glucose levels in diabetic rats with streptozotocin, compared to glibenclamide (5 mg/kg) as a standard, with a statistical significance *p* < 0.001 [48].

### 4.5. Antihyperlipidaemic

An ethanolic flower extract of *A. cathartica* (100, 150, and 300 mg/kg, p.o.) and the compound (**145**) (0.5, 1, and 2 mg/kg, i.p.) decreased the total and High Density Lipoprotein (HDL) cholesterol levels, with significant differences of *p* < 0.001 and *p* < 0.05, respectively, in female Swiss Webster mice at the two highest doses tested [61].

### 4.6. Antifertility

The oral administration of aqueous leaf extracts of *A. cathartica* (150 mg/kg/day for 14, 28, and 42 days) induced infertility and changes in various male reproductive endpoints in Parkes strain mice. Histologically, the testes from the extract-treated mice showed nonuniform degenerative changes in the seminiferous. The treatment also had adverse effects on motility, viability, morphology, and the number of spermatozoa in the cauda epididymides. The fertility of the extract-treated males was also suppressed [72]. The oral administration of (**145**) (15 mg/rat/day for 60 days) in male Wistar rats significantly reduced the weight of the testes, epididymides, seminal vesicles, and prostate compared to the negative controls, and the mobility of the sperm and Sertoli cells also decreased significantly and without systemic side effects. The number of mature Leydig cells was decreased, and a complete suppression of fertility was observed. The content of protein and sialic acid in the testes, epididymides, seminal vesicle, and prostate, as well as the glycogen content of the testes and fructose in the seminal vesicles were reduced. However, testicular cholesterol was elevated [73].

### 4.7. Wound Healing

Aqueous leaf extracts of *A. cathartica* (150 mg/kg/day for 14 days) promoted the wound healing activity in Sprague–Dawley rats. Compared to the controls, treated rats had higher rates of wound contraction, decreased periods of epithelialisation, a higher skin breaking strength, a significantly higher weight of the granulation tissue, and more hydroxyproline content. Histological studies of the granulation tissue in treated rats showed less inflammatory cells and increased collagen formation [8].

### 4.8. Thrombolysis

*A. cathartica* leaves were extracted with methanol and subsequently partitioned with hexane, carbon tetrachloride, chloroform, and water. The thrombolytic activity of the resulting preparation was evaluated in vitro with the concentration of extract at 0.1 mg/100 μL. As a positive control, streptokinase was used. All extracts showed thrombolytic activity with respect to the negative control with a significant difference of *p* < 0.001. The chloroform-partitioned extract presented the highest rate of clot lysis (34.51%) [30].

### 4.9. Purgative Effect

The purgative effect of the aqueous leaf extract of *A. cathartica* was evaluated at different doses (20, 40, 80, 160, and 320 mg/kg orally). As a positive control, the Senna extract was used under the same conditions and the saline solution was used as a negative control; the extract showed a dose-dependent effect [28].

### 4.10. Tyrosinase

The tyrosinase inhibitory activity of the methanol stem powder extracts of *A. cathartica* was examined, and compound (**113**) was identified as having the highest inhibitory activity against tyrosinase (IC_50_: 2.93 μM), which was 15 times stronger than the kojic acid used as a positive control (IC_50_: 43.7 μM) [59].

### 4.11. Amylase

In leaves extracted with ethanol 50% (*v*/*v*), Allotides were identified as being proline-rich and having an α-amylase inhibitory activity [22].

### 4.12. Antiviral

Through an in silico method, it was determined that some compounds present in *A. cathartica* have an antiviral activity against human hepatitis B viral capsid protein [58]. The antirabic activity of methanol and aqueous extracts of leaves was evaluated; however, the extracts did not inhibit the rabies virus at the concentrations evaluated [31]. 

### 4.13. Antimicrobial

The methods most commonly used to evaluate antimicrobial activity are carried out by plaque, disk, and dilution methods. Table 10 describes the different studies carried out with extracts obtained from different parts of *A. cathartica*.

### 4.14. Antimalarial

In an in vivo model in albino rats, the antimalarial activity of a leaf ethanol extract from *A. cathartica* was evaluated at different doses (50, 100, and 200 mg/mL). As a positive control, the compound (**128**) was used (200 mg/kg), and the extract showed an effect similar to (**128**) that was dose-dependent [88].

### 4.15. Nematicide

Bark methanol extracts were evaluated on *Bursaphelenchus xylophilus* (pinewood nematode), where a minimum effective dose (MED) of 5 mg/cotton ball was found [19]. Fractions of hexane extracts of the leaves and stem from *A. cathartica* were evaluated in vitro for nematicidal activity at 0.06, 0.1, and 0.2 mg/mL against juvenile larvae of *Meloidogyne incognita*. The extract showed a nematicidal activity from the first hours of exposure with a rate of 16.87% [89].

### 4.16. Pesticidal

Aqueous extracts of leaves and flowers from *A. cathartica* showed pesticidal properties against *Oligonychus coffeae* [90]. Extractions using petroleum ether, chloroform, and methanol showed pesticidal effects on *Tribolium castaneum* exposed for 24, 48, and 72 h. The LD_50_ values at these time points were 684,376, 319,028, and 225,205 μg/cm^2^ for petroleum ether; 34,289.35, 4,308,567, and 804,082 μg/cm^2^ for chloroform; and 445,092.10, 38,709.10, and 9,906.21 μg/cm^2^ for methanol, respectively [76].

### 4.17. Antihaemorrhagic

Extracts of 96% ethanol made from the leaves, branches, and stems of *A. cathartica* were evaluated for an in vitro haemorrhagic neutralization activity using the blood of a Swiss Webster mouse with 10 μg *Bothrops atrox* venom, and the results obtained showed a neutralization of 72 ± 8%. However, it was not clear if the parts of the plant were evaluated together or separately [12].

### 4.18. Cytotoxicity

The methanolic extract and subsequent fractions (methanol, chloroform, hexane, and carbon tetrachloride) from *A. cathartica* leaves were evaluated for their toxic effects on brine shrimp. The chloroform-, hexane-, and carbon tetrachloride-soluble fractions showed a significant cytotoxic activity against nauplii brine shrimp, with LC_50_ values of 1.45, 5.00, and 5.24 μg/mL, respectively [30]. The methanol and aqueous extracts of leaves at concentrations of 10, 5, 2.5, 1.25, and 0.6 mg/mL did not show a cytotoxic activity on BHK-21 cells [31]. In another study of methanol extracts from leaves, an IC_50_ of 85 μg/mL was found for P388 leukaemia cells [86]. The use of silver nanoparticles (AgNO_3_) with aqueous latex extracts of *A. cathartica* showed a dose-dependent effect against human mononuclear blood cells [91]. The methanol, ethyl acetate, petroleum ether, and chloroform extracts from leaves of *A. cathartica* showed LD_50_ values of 111.61, 131.14, 332.42, and 47.86 μg/mL, respectively, against *Artemia salina* [76]. Compounds (**142**), (**139**), and (**138**) obtained from 95% ethanol leaf extracts showed a significant tumour suppression in vitro against human nasopharnyx carcinoma (KB) cells with an LD_50_ of 2.1, 2.6, and 2.7 μg/mL, respectively [65].

### 4.19. Antioxidants

The antioxidant activity of *A. cathartica* was evaluated in vitro using the FRAP and TEAC methods with Methanol:Acetic acid:Water extracts (50:3.7:46.3 *v*/*v*/*v*) as well as the water-soluble and fat-soluble fraction from flowers, which showed antioxidant activities via FRAP of 18.95 ± 0.34 and 4.56 ± 0.11 μmol Fe (II)/g, respectively. By the TEAC method, the antioxidant activity was 7.35 ± 0.26 and 1.46 ± 0.21 μmol Trolox/g, respectively [24]. The ethanol extracts from the leaves had an antioxidant activity (based on the DPPH method) that was dose-dependent at concentrations of 0.5, 1, 2, and 5 mg/mL [92]. The methanol extracts from the flowers showed an antioxidant activity by the DPPH method at a concentration of 0.6 mg/mL [93]. Different plant parts were analysed for their antioxidant activity in vitro where it was higher in shoot > root > leaves > flowers. The relative peroxidase and superoxide dismutase (SOD) activities were in the order of root > shoot > leaves > flowers [17]. The relative in vitro antioxidant activity of various leaf extracts of *A. cathartica* was in the following order: butylated hydroxyl toluene (BHT) > Dia-Ion resin Absorbed > Chloroform > Ethyl acetate (EtOAc) > Methanol (MeOH) > Petroleum ether (PE) [76]. The carbon tetrachloride fraction from a methanol extract from the leaves had an IC_50_ of 47.5 ± 0.11 μg/mL in the DPPH model [69]. In the study of isolated compounds, (**145**) (100 mg/kg orally) administered to female Swiss mice significantly decreased the levels of lipid hydroperoxides (LOOH) and reduced the glutathione (GSH) levels and SOD activity, whereas the catalase (CAT) activity remained unchanged compared with the untreated group. The standard drug 5-ASA reduced the LOOH content and increased the SOD activity compared to the vehicle (VEH) group, whereas treatment with (**145**) promoted a complete improvement of the oxidative unbalance, restoring all the parameters [71]. In an in vivo model using albino rats, the antioxidant activity of the ethanol extract of leaves (50, 100, and 200 mg/mL) was evaluated, and as a positive control, the compound (**128**) was used (200 mg/kg), showing a significant increase in TBARS, with a decrease in GSH and CAT levels [88].

## 5. Toxicity

*A. cathartica* is reported to be a venomous plant due to the presence of a cardiotoxic glycoside [25]. All parts of the plant cause dermatitis [29]. It has been reported that the leaves and sap produce persistent diarrhoea with high consumption rates. Also, skin irritation has been reported, but the responsible compounds have not been identified [3]. Studies have been carried out on the cytotoxicity and genotoxicity of hexane extracts of leaves of *A. cathartica*. It was demonstrated that a concentration of 315 mg/mL is cytotoxic to lymphocytes with a 79% cellular viability. In HeLa cells, an IC_50_ of 13.5 mg/mL was found. These results showed a genotoxicity (*p* < 0.01) for both cell types, which led the authors to suggest that *A. cathartica* not be used as a medicinal plant [94]. However, it is necessary to standardize the HPLC samples for at least one compound present in the plant. In the evaluation of acute toxicity (i.p.) in mice, it was observed that the LD_50_ was 1320 ± 15 mg/kg [28]. The oral administration of 2 mg/kg of ethanolic extract of flowers and the compound (**145**) in Swiss Webster mice administered as a single dose and evaluated at 14 days showed no toxic effects, no changes in biochemical or haematological parameters, and no genotoxic effects [61]. The toxicological evaluation of the petroleum ether extract of leaves in albino mice showed no toxicity at doses of 100 to 1000 mg/kg p.o. for 72 h [81].

## 6. Biotechnological Use

The effects of 2,4-dichlorophenoxyacetic acid (2,4-D) and 6-benzylaminopurine (BAP) on the induction of callus from leaf and stem explants were investigated. The regeneration of plants from the nodal explants was achieved. The explants were cultured in a Murashige and Skoog (MS) medium, supplemented with different concentrations of 2,4-D (0.5 and 1.0 mg/L) or in combinations of 2,4-D (0.5, 1.0, and 1.5 mg/L) with BAP (0.5, 1.0, and 1.5 mg/L). In the study of plant regeneration, the nodal explants were cultivated in an MS medium supplemented with BAP at 1.0, 3.0, or 5.0 mg/L for the multiplication of shoots. The MS basal medium was used as a control and was also used for the elongation of the shoots. All cultures were incubated under a photoperiod of 16 h of light and 8 h of darkness. For callus induction, the explants of leaves and stems grown at 1.0 mg/L of 2,4-D and 1.0 mg/L of BAP gave the best callus response (100%). For the multiplication of shoots, the MS medium supplemented with 5 mg/L of BAP gave the best response (100%) with multiple buds formed [46].

## 7. Conclusions

This review details the ethnomedical, phytochemical, pharmacological, toxicological, and biotechnological uses of *A. cathartica*. Although there have been several studies on the pharmacological activity of *A. cathartica*, the potential of this plant is as an analgesic, anti-inflammatory, antidepressant, antidiabetic, antihyperlipedaemic, antifertility agent, wound healing, trombolytic, purgative, tyrosine, amylase, antimicrobial, antimalarial, nematicide, antioxidant, etc. agent. 

## Figures and Tables

**Table 1:** The hydrocarbons from *A. cathartica*.

No.	Compound’s Name	Parts Used	Reference
(**1**)	*n*-Heneicosane	Flowers	[10]
(**2**)	*n*-Tricosane	Flowers	[10]
(**3**)	*n*-Pentacosane	Flowers	[10]

**Table 2:** The alcohols, esters, ethers, aldehydes, and ketones from *A. cathartica*.

No.	Compound’s Name	Parts Used	Reference
(**4**)	1-Hexanol	Flowers	[10]
(**5**)	1-Hexadecanol	Flowers	[10]
(**6**)	Glycerin	Leaves and stem	[57]
(**7**)	(*Z*)-3-Hexenol	Flowers	[10]
(**8**)	Nerol	Flowers	[35]
(**9**)	Geraniol	Flowers	[35]
(**10**)	(*E*)-Nerolidol	Flowers	[35]
(**11**)	Hexanoic acid, ethyl ester	Leaves and stem	[57]
(**12**)	Octanoic acid, ethyl ester	Leaves and stem	[57]
(**13**)	Decanoic acid, ethyl ester	Leaves and stem	[57]
(**14**)	Hexadecanoic acid, ethyl ester	Leaves and stem	[57]
(**15**)	Octadecanoic acid, ethyl ester	Leaves and stem	[57]
(**16**)	Nonadecanoic acid, ethyl ester	Leaves	[57]
(**17**)	9,12-Octadecadienoic acid, ethyl ester	Leaves and stem	[57]
(**18**)	9,12,15-octadecatrienoic acid, ethyl ester, (*Z*,*Z*,*Z*)-	Leaves and stem	[43,57]
(**19**)	Methyl linoleate	Flowers	[10]
(**20**)	Propane, 1,1,3-triethoxy-	Leaves and stem	[57]
(**21**)	Hexanal	Flowers	[10]
(**22**)	Heptanal	Flowers	[10]
(**23**)	Octanal	Flowers	[10]
(**24**)	(*E*)-2-Heptenal	Flowers	[10]
(**25**)	*Cis*,*cis*,*cis*-7,10,13-hexadecatrienal	Leaves	[57]
(**26**)	2-furancarboxaldehyde, 5-(hydroxymethyl)-	Stem	[57]
(**27**)	6,10,14-Trimethyl-2-pentadecanone	Flowers	[10]

**Table 3:** The fatty acids and phospholipids from *A. cathartica*.

No.	Compound’s Name	Parts Used	Reference
(**28**)	Dodecanoic acid	Flowers, leaves, and stem	[52,57]
(**29**)	Tetradecanoic acid	Flowers, leaves, and stem	[7,52,57]
(**30**)	Pentadecanoic acid	Leaves and flowers	[7,57]
(**31**)	Hexadecanoic acid	Flowers, leaves, and stem	[7,43,52,57]
(**32**)	Heptadecanoic acid	Flowers	[7]
(**33**)	Octadecanoic acid	Flowers and leaves	[7,52]
(**34**)	Nonadecanoic acid	Flowers	[7]
(**35**)	Eicosanoic acid	Flowers and leaves	[7,52]
(**36**)	Heneicosanoic acid	Flowers	[7]
(**37**)	Docosanoic acid	Flowers	[7]
(**38**)	Tetracosanoic acid	Flowers	[7]
(**39**)	Pentacosanoic acid	Flowers	[7]
(**40**)	Hexacosanoic acid	Flowers	[7]
(**41**)	2-Hydroxyhexadecanoic acid	Flowers	[7]
(**42**)	2-Hydroxyoctadecanoic acid	Flowers	[7]
(**43**)	2-Hydroxyeicosanoic acid	Flowers	[7]
(**44**)	2-Hydroxydocosanoic acid	Flowers	[7]
(**45**)	2-Hydroxytricosanoic acid	Flowers	[7]
(**46**)	2-Hydroxytetracosanoic acid	Flowers	[7]
(**47**)	2-Hydroxydocosenoic acid	Flowers	[7]
(**48**)	2-Hydroxytetracosenoic acid	Flowers	[7]
(**49**)	7-Eicosenoic acid	Flowers	[7]
(**50**)	9-Hexadecenoic acid	Flowers	[7]
(**51**)	9-Octadecenoic acid	Flowers, leaves, and stem	[7,52,57]
(**52**)	9-Nonadecenoic acid	Flowers	[7]
(**53**)	11-Octadecenoic acid	Flowers	[7]
(**54**)	11-Eicosenoic acid	Flowers	[7]
(**55**)	13-Eicosenoic acid	Flowers	[7]
(**56**)	13-Docosenoic acid	Flowers	[7]
(**57**)	15-Docosenoic acid	Flowers	[7]
(**58**)	5,9-Nonadecadienoic acid	Flowers	[7]
(**59**)	17-Methyl-5,9-octadecadienoic acid *	Flowers	[7]
(**60**)	11,14-Eicosadienoic acid	Flowers	[7]
(**61**)	9,12-Octadecadienoic acid	Flowers and leaves	[7,52]
(**62**)	9,12-Octadecadienoic acid (*Z*,*Z*)-	Stem	[57]
(**63**)	9,12,15-Octadecatrienoic acid	Flowers	[7]
(**64**)	9,12,15-Octadecatrienoic acid (*Z*,*Z*,*Z*)-	Leaves and Stem	[44,57]
(**65**)	Phosphatidylinositol	Flowers	[7]
(**66**)	Phosphatidycholine	Flowers	[7]

Note: * Not reported in nature.

**Table 4:** The volatile compounds from *A. cathartica*.

No.	Compound’s Name	Parts Used	Reference
(**67**)	(*E*)-β-ocineme	Flowers	[10]
(**68**)	(*E*)-β-Farnesene	Flowers	[10]
(**69**)	(*E*,*E*)-α-Farnesene	Flowers	[10]
(**70**)	(*Z*)-β-ocimene	Flowers	[10]
(**71**)	(*E*,*E*)-Geranyl linaool	Flowers	[10]
(**72**)	(*Z*,*Z*)-Farnesol	Flowers	[10]
(**73**)	1-Octen-3-ol	Flowers	[10]
(**74**)	2-Butooxyethanol	Flowers	[10]
(**75**)	1,8-cineole	Flowers	[10]
(**76**)	2-Phenylethanol	Flowers	[10]
(**77**)	Benzaldehyde	Flowers	[10]
(**78**)	Benzoic acid, 2-hydroxy-, methyl ester	Leaves	[57]
(**79**)	Benzyl isothiocyanate	Flowers	[35]
(**80**)	Phenylacetonitrile	Flowers	[35]
(**81**)	Bicyclogermacrene	Flowers	[35]
(**82**)	Trans-Linalool oxide	Flowers	[35]
(**83**)	Cis-sabinehydrate	Flowers	[10]
(**84**)	Germacrene D	Flowers	[35]
(**85**)	Indole	Flowers	[10]
(**86**)	Linalool	Flowers	[35]
(**87**)	Myrcene	Flowers	[10]
(**88**)	Limonene	Flowers	[10]
(**89**)	γ-Terpinene	Flowers	[10]
(**90**)	α-Terpinene	Flowers	[10]
(**91**)	*p*-cyneme	Flowers	[10]
(**92**)	Terpinolene	Flowers	[10]
(**93**)	α-Terpineol	Flowers	[10,35]
(**94**)	Terpinen-4-ol	Flowers	[10]
(**95**)	3,7,11,15-tetramethyl-2-hexadecen-1-ol	N.R.	[58]
(**96**)	Cumin alcohol	Flowers	[35]
(**97**)	Phenylacetaldehyde	Flowers	[10,35]
(**98**)	α-Thujene	Flowers	[10]
(**99**)	α-Copaene	Flowers	[35]
(**100**)	α-Cubebene	Flowers	[35]
(**101**)	β-Cubebene	Flowers	[35]
(**102**)	δ-Cadinene	Flowers	[35]
(**103**)	α-Humulene	Flowers	[35]
(**104**)	α-Pinene	Flowers	[10]
(**105**)	β-Pinene	Flowers	[10]
(**106**)	Camphene	Flowers	[10]
(**107**)	Isoborneol	Flowers	[10]
(**108**)	β-Caryophyllene	Flowers	[10,35]
(**109**)	β-Elemene	Flowers	[35]

Note: N.R. = Not reported.

**Table 5:** The phenolic compounds and flavonoids from *A. cathartica*.

No.	Compound’s Name	Parts Used	Reference
(**110**)	Protocatechuic acid	Flowers	[24]
(**111**)	Gallic acid	Flowers	[24]
(**112**)	1-(3-(4-Allyl-2,6-dimethoxyphenoxy)-4-methoxyphenyl)propane-1,2,diol	Stem	[59]
(**113**)	Glabridin	Stem	[59]
(**114**)	2-phenanthrenecarboxaldehyde, 1,2,3,4,4a,4b,5,6,7,8,8a,9-dodecahydro-7-hydroxy-2,4b,8,8-tetramethyl-	Leaves and stem	[57]
(**115**)	Epicatechin	Flowers	[24]
(**116**)	Naringenin	Stem	[59]
(**117**)	Kaempferol	Stem	[59]
(**118**)	Quercetin	Flowers	[60]
(**119**)	Quercitrin	Flowers	[60]
(**120**)	Rutin	Flowers	[61]

**Table 6:** The alkaloids from *A. cathartica*.

No.	Compound’s name	Parts Used	Reference
(**121**)	6,7-dimethylthieno(2,3-b) quinolin-3-ylamine	Stem	[57]
(**122**)	Heptanediamide, *N*,*N′*-di-benzoyloxy-	Stem	[57]

**Table 7:** The steroids and terpenes from *A. cathartica*.

No.	Compound’s Name	Parts Used	Reference
(**123**)	β-sitosterol	Leaves and stem	[63]
(**124**)	β-Amyrin	Leaves and stem	[63]
(**125**)	Ursolic acid	Leaves and stem	[14,63]
(**126**)	Phytol	Flowers, leaves, and stem	[10,57]
(**127**)	Squalene	Leaves	[57]
(**128**)	Vitamine E	Leaves	[57]
(**129**)	Zeaxanthin	Flowers	[21]
(**130**)	b-Carotene	Flowers	[21]
(**131**)	Lutein	Flowers	[21]
(**132**)	Neoxanthin	Flowers	[21]
(**133**)	Violaxanthin	Flowers	[21]

**Table 8:** The lactones from *A. cathartica*.

No.	Compound’s Name	Parts Used	Reference
(**134**)	4H-Pyran-4-one, 2,3-dihydro-3,5-dihydroxy-6-methyl-	Leaves and stem	[57]
(**135**)	Vitamine C	Leaves	[14]
(**136**)	Dendrolasin	Flowers	[35]
(**137**)	Allamandin	Root bark	[65]
(**138**)	Plumericin	Leaves, root, stem, leaves, flowers, bark, and root bark	[9,18,65,66]
(**139**)	Isoplumericin	Leaves, root, root bark, stem, and bark	[9,18,65,66]
(**140**)	Acetylallamandin	Root bark	[65]
(**141**)	Allamdin	Root bark	[65]
(**142**)	Allamandicin	Root bark	[65]
(**143**)	Penta-acetylplumieride coumarate	Root	[66]
(**144**)	Octa-acetylplumieride coumarate	Root	[66]
(**145**)	Plumieride	Root, stem, leaves, flowers, bark, and bark (inner part)	[18]
(**146**)	Plumieride coumarate	Root, stem, leaves, flowers, bark, and bark (inner part)	[18,66]
(**147**)	Plumieride coumarate glucoside	Root, stem, leaves, flowers, bark, and bark (inner part)	[18,66]

**Table 9:** The carbohydrates from *A. cathartica*.

No.	Compound’s Name	Parts Used	Reference
(**148**)	1-Deoxy-d-mannitol	Leaves	[57]
(**149**)	3-*O*-methyl-d-glucose	Leaves and stem	[43,57]
(**150**)	Glucose	Nectar	[67]
(**151**)	Rhamnose	Nectar	[15]
(**152**)	Fructose	Nectar	[67]
(**153**)	β-l-arabinopyranoside, methyl	Leaves	[57]

**Table 10:** The effect of *A. cathartica* extract on a microorganism.

Microorganism	Used Part	Extract/Fraction	Reference
**Gram Positive**			
*Agrobacterium tumefaciens*	Flowers and leaves	Bound and free flavonoids, steroids, and alkaloids	[74]
*Bacillus cereus*	Leaves	TCM	[75]
		EtOAc	[69]
		MeOH, PE, TCM, EtOAc, and Dia-Ion	[76]
*Bacillus megaterium*	Leaves	TCM	[75]
		EtOAc	[69]
*Bacillus subtilis*	Flowers and Leaves	Bound and free flavonoids and steroids	[74]
	Leaves	TCM	[75]
		Water *	[77]
*Sarcina lutea*	Leaves	TCM	[75]
*Staphylococcus aureus*	Flowers	Water *	[78]
		MeOH 90%	[79]
	Flowers and leaves	Free flavonoids, alkaloids, bound flavonoids, and steroids	[74]
	Leaves	MeOH, PE, TCM, EtOAc, and Dia-Ion	[76]
		TCM	[77]
	Root	MeOH, EtOAc, and PE	[80]
	All plant	N.E.	[68]
*Staphylococcus aureus* **	Leaves	MeOH, EtOH, EtOAc, TCM, and PE	[81]
*Streptococcus pneumonia*	Root	MeOH, EtOAc	[80]
**Gram Negative**			
*Acinetobacter baumannii* **	Flowers	EtOH	[82]
Acinetobacter sp **	Leaves	MeOH, EtOH, EtOAc, Water, and PE	[81]
*Bacillus subtillis*	Leaves	Bound flavonoids	[74]
*Escherichia coli*	Flowers	Water *	[78]
	Flowers and leaves	Bound flavonoids and steroids	[74]
	Flowers	MeOH 90%	[79]
	Leaves	TCM	[75]
		MeOH, PE, TCM, EtOAc, and Dia-Ion	[76]
	Root	EtOAc	[80]
*Escherichia coli* **	Leaves	Water and PE	[81]
		Water	[32]
*Klebsiella pneumoniae*	Root	MeOH and EtOAc	[80]
	Flowers	Water *	[78]
	Flowers and leaves	Bound and free flavonoids	[74]
	Leaves	Water *	[77]
*Klebsiella pneumoniae* **	Leaves	Water	[32]
*Proteus mirabilis* **	Leaves	Water	[32]
Proteus sp **	Leaves	PE	[81]
*Proteus vulgaris*	Leaves	MeOH, PE, TCM, EtOAc, and Dia-Ion	[76]
*Pseudomonas aeruginosa*	Leaves	TCM	[75]
		Water *	[77]
*Pseudomonas aeruginosa* **	Leaves	Water	[32]
		MeOH, EtOAc, TCM, and PE	[81]
*Salmonella paratyphi*	Leaves	TCM	[75]
		EtOAc	[69]
*Salmonella typhi*	Leaves	TCM	[75]
		EtOAc	[69]
*Salmonella typhimurium*	Flowers	Water *	[78]
*Shigella boydii*	Leaves	TCM	[75]
*Shigella dysenteriae*	Leaves	TCM	[75]
*Vibrio mimicus*	Leaves	TCM	[75]
*Vibrio parahemolyticus*	Leaves	TCM	[75]
**Fungi**			
*Aspergillus flavus*	Leave and Flowers	MeOH	[83]
*Aspergillus flavus*	Leaves	MeOH:Water (2:1 *v*/*v*)	[84]
		Water *	[77]
*Aspergillus niger*	Leaves	TCM	[75]
		Water *	[77]
*Candida albicans*	Leaves	EtOH 99.8%	[85]
		TCM	[75]
		MeOH	[34]
	Leave and Flowers	MeOH	[83]
	Flowers	MeOH 90%	[79]
Candida albicans **	Leaves	EtOH	[81]
*Carvularia lunata*	Leaves	PE and TCM	[40]
*Epidermophyton floccosum*	Leaves	MeOH	[86]
*Microsporum gypseum*	Leaves	MeOH	[86]
*Pityrosporum ovale*	Leaves	EtOH 99.8%	[85]
*Sacharomyces cerevaceae*	Leaves	TCM	[75]
**Plant Fungi**			
*Colletotrichum gloeosporioides*	Leaves	TCM	[42]
*Colletotrichum lidemuthianum*	Leaves	PE and TCM	[40]
*Curvularia luunata*	Leaves	Water *	[77]
*Fusarium oxysporum*	Leaves	PE and TCM	[40]
		MeOH, EtOH, EtOAc, and EtOH 50%	[87]
*Fusarium oxysporum f.sp. capsici*	Leave	MeOH	[16]
*Phomopsis vexans*	Leaves	MeOH, EtOH, EtOAc, and EtOH 50%	[87]
*Phytophthora capsici*	Leaves	MeOH, EtOH, EtOAc, and EtOH 50%	[87]
*Rhizopus arrhizus*	Leaves	Water *	[77]
*Rhizotonia solani*	Leaves	MeOH, EtOH, EtOAc, and EtOH 50%	[87]
*Sclerotium rolsfsii*	Leaves	MeOH, EtOH, EtOAc, and EtOH 50%	[87]

Note: * Used with silver nanoparticles (AgNPs), ** Clinical isolates, TCM = Chloroform, PE = Petroleum ether, MeOH = Methanol, EtOH = Ethanol, and EtOAc = Ethyl acetate.
